# Effects of Extrusion Process Variables on the Physicochemical and Textural Properties of Isolated Rice Protein-Enhanced Low-Moisture Meat Analogs

**DOI:** 10.3390/foods15152754

**Published:** 2026-08-05

**Authors:** Yu Zhang, Joo-Won Kim, Hyerim Jeon, Gi-Hyung Ryu, Bon-Jae Gu, Da-Eun Jung

**Affiliations:** 1 Department of Food and Quality Engineering, Nanning University, Nanning 530200, China; 2 Department of Food Science and Technology, Food and Feed Extrusion Research Center, Kongju National University, Yesan 32439, Republic of Korea; 3 Department of Food and Resource Economics, Dankook University, Cheonan 31116, Republic of Korea

**Keywords:** alternative protein, textured vegetable protein, process optimization, cereal protein blend, water-holding capacity, composite quality index

## Abstract

In this study, the physicochemical and textural properties of isolated rice protein-enhanced low-moisture meat analogs (IRPE-LMMAs) were systematically evaluated in relation to key extrusion variables. A dry blend containing 10% isolated rice protein was processed by low-moisture extrusion cooking, and moisture content, barrel temperature, and screw speed were varied using a Box–Behnken response surface design. The quality characteristics of IRPE-LMMAs were assessed based on appearance, water-holding capacity, integrity index, nitrogen solubility index, springiness, cohesiveness, chewiness, and cutting strength. Among the tested variables, moisture content showed the most apparent influence on both hydration-related and texture-related properties. Higher moisture content tended to increase water-holding capacity, nitrogen solubility index, springiness, and cohesiveness, whereas chewiness and cutting strength decreased. These results indicate that increased moisture content improved water-related functionality and elastic properties but reduced firmness-related textural attributes. Barrel temperature and screw speed showed response-dependent effects, suggesting that their influence should be interpreted in combination with moisture content and the specific quality parameter considered. Response surface methodology based on chewiness identified a practical processing window within the experimental range of 39–40% moisture content, 141–144 °C barrel temperature, and 205–215 rpm screw speed. Supplementary exploratory regression analysis suggested that moisture content was a major contributor to the composite quality index, while firmness-related responses required separate consideration. Overall, the results indicate that isolated rice protein can be incorporated into LMMA formulations, and that process optimization should focus on balancing hydration, elasticity, and firmness according to the intended product quality.

## 1. Introduction

Meat alternatives are developed to reproduce the quality attributes of conventional meat using alternative protein sources, including plant proteins [1], fungal proteins [2], or cultured cells [3] to provide meat-like appearance, texture, and nutritional value [4]. Recently, interest in meat alternatives has continued to grow as global demand for dietary protein increases and consumers become more aware of health, environmental sustainability, and animal welfare issues [5]. From a health perspective, plant-based meat alternatives may provide a useful option for consumers seeking to reduce meat intake because they contain no cholesterol and generally lower levels of saturated fat than conventional meat products [6]. Additionally, their development aligns with environmental efforts to reduce the ecological burdens of livestock production, such as greenhouse gas emissions and land use [7]. Therefore, creating structured plant-based foods with acceptable quality remains a key research focus in food processing.

Soy protein is widely used as a primary matrix derived from plants for meat analogs due to its texturizing performance during extrusion. Previous studies have shown that soy protein, often in combination with wheat gluten or other protein ingredients, can be used to prepare textured vegetable protein and meat analog products with improved processing stability and meat-like characteristics [8]. However, soy-based formulations still have several limitations that may restrict their broader application. For example, soy protein can generate beany or grassy off-flavors associated with lipoxygenase activity [9]. In addition, allergen concerns and the relatively low methionine content of soy protein remain important formulation challenges [10]. Rice protein has therefore received attention as a complementary plant protein source for meat analog development. However, compared to soy, rice protein is less allergenic, has a milder taste, and is associated with cholesterol-lowering benefits [11]. From a nutritional perspective, rice protein can complement the methionine limitation of soy protein, whereas soy protein can compensate for the relatively low lysine content of rice protein [12]. These complementary properties suggest that the incorporation of isolated rice protein into soy-based meat analog formulations may improve ingredient diversity and provide a useful strategy for designing plant protein blends with balanced nutritional and processing characteristics.

Low-moisture extrusion cooking (LMEC) is widely used to generate structured plant protein products and low-moisture meat analogs because it can transform protein-rich formulations into structured matrices by utilizing the simultaneous application of high temperature, pressure, and mechanical shear in conditions of restricted moisture [4,13,14]. During extrusion, plant proteins undergo denaturation, unfolding, aggregation, and network formation, and these structural changes strongly influence the expansion behavior, internal morphology, hydration properties, and texture of the final extrudates. Among the processing variables involved in LMEC, feed moisture content, barrel temperature, and screw speed are particularly important because they determine the balance between thermal energy input, shear intensity, protein transformation, melt viscosity, and structural retention. Changes in these variables may improve certain quality attributes while reducing others; therefore, process optimization should consider multiple physicochemical and textural responses rather than relying on a single processing factor. This is especially important when a new protein ingredient is introduced into a meat analog formulation, because different plant proteins may respond differently to extrusion conditions. Although various plant proteins have been investigated for extrusion, the specific interactive effects of low-moisture extrusion process variables on the structural network development and multiple physical quality attributes of isolated rice protein integrated into a soy protein matrix remain unexplored. Understanding these distinct processing behaviors represents a scientific niche necessary to optimize blended plant protein systems. In this study, isolated rice protein-enhanced low-moisture meat analogs containing 10% IRP were prepared by LMEC. This study investigated the impact of moisture content, barrel temperature, and screw speed on the physical, textural, and functional properties of the samples, including appearance, water-holding capacity, integrity index, nitrogen solubility index, springiness, cohesiveness, chewiness, and cutting strength. In addition, response surface methodology was applied to identify practical processing conditions for producing isolated rice protein-enhanced LMMAs within the experimental range.

## 2. Materials and Methods

### 2.1. Materials

The dry blends for formulating the isolated rice protein-enhanced low-moisture meat analogs (IRPE LMMAs) consisted of isolated rice protein (IRP), isolated soy protein (ISP), wheat gluten (WG), and corn starch (CS). The specific sources of these ingredients were as follows: IRP was provided by Vedan Vietnam Enterprise Co., Ltd. (Dong Nai City, Vietnam); ISP was obtained from Pingdingshan Tianjing Plant Albumen Co., Ltd. (Pingdingshan, China); WG was sourced from Roquette Freres (Lestrem, France); and CS was supplied by Samyang Ltd. (Ulsan, Republic of Korea). These components were blended into a dry formulation according to a ratio of 10:40:40:10 (IRP:ISP:WG:CS). This specific formulation ratio was selected based on previous studies [15,16] and further established through preliminary experiments conducted to ensure adequate protein network development and optimal structural integrity. Specifically, IRP contained 4.8% moisture, 82.9% crude protein, 4.1% crude fat, 1.5% crude ash, and 6.7% carbohydrate. ISP consisted of 6.9% moisture, 80.9% crude protein, 2.3% crude fat, 5.1% crude ash, and 4.8% carbohydrate. WG was composed of 9.5% moisture, 72.8% crude protein, 4.0% crude fat, 0.8% crude ash, and 12.9% carbohydrate. Additionally, the nitrogen solubility index was found to be 82.08% for IRP, 95.53% for ISP, and 93.81% for WG [15].

### 2.2. Low-Moisture Extrusion Process

**Figure 1:**
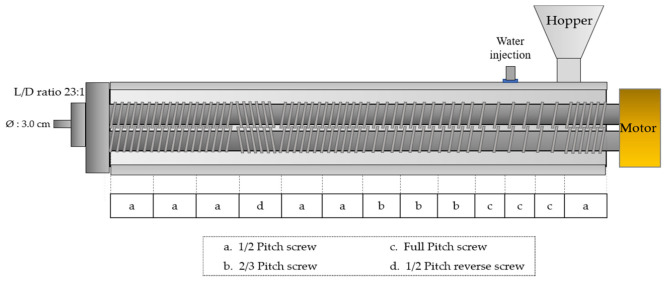
Screw configuration for low-moisture extrusion cooking using the twin-screw extruder. Reproduced from [14], licensed under CC BY 4.0.

The low-moisture extrusion process was performed utilizing a co-rotating intermeshing twin-screw extruder (THK 31T, Incheon Machinery Co., Ltd., Incheon, Republic of Korea), as illustrated in Figure 1. The equipment was characterized by a screw diameter of 3 cm and a length of 69 cm, which yields an L/D ratio of 23:1. Extrudates were molded through a short-slit die (1 × 0.45 × 8 cm; W × H × L). The dry ingredients were thoroughly mixed and supplied to the extruder without external preconditioning. Water was continuously supplied using a metering pump, and the water flow rate was adjusted to achieve the target feed moisture contents of 30, 35, and 40%. The barrel temperature profile from the feeding zone to the final barrel zone was set at 60 °C, 100 °C, and 140, 150, or 160 °C, respectively. The experimental setup was formulated utilizing a Box–Behnken response surface design, which incorporated three independent variables: moisture content (X_1_), barrel temperature (X_2_), and screw speed (X_3_). Each factor was assessed at three distinct levels (Table 1). This experimental design included three center point replicates performed in a randomized order to provide the experimental degrees of freedom required to estimate pure error and calculate the lack of fit analysis for model validation. During the operation, the feed rate was held steady at 100 g/min, whereas experimental variables such as moisture content, barrel temperature, and screw speed were varied as specified in the experimental design (Table 1). Post-extrusion, the specimens were subjected to drying at 50 °C for 12 h. A subset of these dried extrudates was milled using a stainless-steel mixer and subsequently fractionated through 70-mesh and 50-mesh sieves.

### 2.3. Water Holding Capacity (WHC)

The water-holding capacity (WHC) was performed following a modified method previously described by Zhang et al. [17]. A 5 g portion of the dried IRPE-LMMAs (dry basis) was combined with 100 mL of distilled water and subsequently hydrated in a thermostatic water bath maintained at 90 °C for a duration of 30 min. After rehydration, the sample was drained on a 20-mesh sieve for 15 min to eliminate excess unbound water. WHC was determined from the variation in mass between the dried and rehydrated samples using Equation (1). Data were obtained from triplicate measurements, and the mean values were used for the analysis.

(1) WHC (%) = (M_2_ − M_1_)/M_1_ × 100%

where M_1_ is the weight of dried sample, and M_2_ is the weight of rehydrated sample.

### 2.4. Integrity Index

The integrity index was measured according to the procedure reported by Samard and Ryu [18], with slight adjustments. The hydration process was conducted by placing 5 g (dry basis) of the dried IRPE-LMMA sample into an Erlenmeyer flask with 100 mL of distilled water, which was then maintained at 80 °C in a water bath for a duration of 30 min. The hydrated sample was then sterilized using an autoclave (PAC-60, Lab House Co., Pocheon-si, Republic of Korea). After sterilization, the sample was transferred to a 20-mesh sieve and washed with running water for 1 min. The retained material was dispersed in 100 mL of distilled water using an Ultra-Turrax homogenizer (T10 Basic Ultra-Turrax, IKA Co., Breisgau, Germany) operating at 17,450 rpm for 1 min, followed by washing again through the same sieve for 1 min. The residual sample was dried at 105 °C until a constant weight was achieved. The integrity index was subsequently determined using Equation (2), with all analyses conducted in triplicate to ensure reproducibility.

(2) Integrity index/% = W_b_/W_a_ × 100

where W_a_ is the dry weight of the sample before sterilization, and W_b_ is the dry weight of the residue after sterilization, dispersion, washing, and drying.

### 2.5. Nitrogen Solubility Index (NSI)

The nitrogen solubility index (NSI) was quantified by applying a modified version of the protocol proposed by Samard et al. [19]. For the extraction of soluble nitrogen, a 0.2 g portion of ground IRPE-LMMA was dispersed into 10 mL of a 0.5% KOH solution. This mixture was agitated at 120 rpm and 30 °C for 30 min using an orbital shaker (H-1000-3, Hanil Science Industrial, Gimpo-si, Republic of Korea). Following centrifugation at 3000 rpm for 3 min, the supernatant was collected for subsequent colorimetric assessment. The extracted supernatant was mixed with a ninhydrin reagent prepared using ninhydrin (Sigma-Aldrich, St. Louis, MO, USA) in an ethylene glycol–sodium acetate solvent system, followed by incubation at 100 °C for 10 min. After an appropriate dilution step, absorbance values were recorded at 575 nm using a spectrophotometer (UL 61010-1, MET Laboratories, Inc., Baltimore, MD, USA), with distilled water employed as the blank reference. To determine total nitrogen, a 0.2 g aliquot of the ground sample underwent hydrolysis in 5 mL of 6 N HCl at 100 °C for 24 h. The resulting hydrolysate was then diluted with distilled water and processed through the identical centrifugation and colorimetric steps outlined previously. Finally, the NSI was derived utilizing the formula presented in Equation (3).

(3) NSI/% = Soluble nitrogen content/Total nitrogen content × 100

### 2.6. Texture Profile Analysis (TPA) and Cutting Strength

The textural properties, including Texture Profile Analysis (TPA) and cutting strength, were evaluated via a Sun rheometer (Compac-100, Sun Science Co., Tokyo, Japan). For the TPA assessment, IRPE-LMMA specimens were shaped into 1 × 1 × 1 cm cubes and subjected to compression using a 25 mm diameter cylindrical probe. The assay was conducted at a crosshead speed of 100 mm/min, utilizing a 10 kg load cell and maintaining a 20 mm clearance between the sample supports. Key parameters such as springiness, cohesiveness, and chewiness were derived through Equations (4)–(6). Regarding the cutting strength analysis, samples were prepared in 1 × 1 × 1.5 cm dimensions. Both vertical and parallel cutting resistances were recorded using a specialized cutting probe (7.5 × 38.3 mm), and the values were calculated according to Equation (7). Every textural parameter was measured in ten replicates, with data presented as the mean value ± standard deviation.

(4) Springiness (%) = D_2_/D_1_ × 100

where D_1_ is the distance at the first maximum stress, and D_2_ is the distance at the second maximum stress.

(5) Cohesiveness (%) = A_2_/A_1_ × 100

where A_1_ is the area under the first compression curve, and A_2_ is the area under the second compression curve.

(6) Chewiness (g) = Springiness × Cohesiveness × Maximum stress/10,000

(7) Cutting strength (g/cm^2^) = Maximum stress/Cross-sectional area

### 2.7. Experiment Design and Data Analysis

The extrusion process was optimized using a 15-run Box–Behnken design with three center points via Design Expert software version 8.0.6. The independent variables evaluated at three levels were moisture content (X_1_), barrel temperature (X_2_), and screw speed (X_3_). Chewiness was designated as the primary response variable for response surface optimization because it is a representative textural attribute related to the eating quality of low-moisture meat analogs. The remaining physicochemical and textural properties were evaluated individually to characterize the effects of the extrusion process variables rather than to establish a combined optimization objective.

To determine the statistical validity of the fitted quadratic polynomial model, which consists of linear, interaction, and quadratic terms, an analysis of variance (ANOVA) was conducted. The significance of the model was evaluated by examining F-values and *p*-values at thresholds of *p* < 0.05 and *p* < 0.01. Contour plots were derived from the regression model to illustrate the impact of extrusion variables on chewiness.

IBM SPSS Statistics 22.0 (IBM, Armonk, NY, USA) was employed for data analysis, utilizing Duncan’s multiple range test to differentiate treatment means at a significance level of *p* < 0.05. To augment the response surface optimization based on chewiness, exploratory multiple regression analyses were performed to gauge the relative influence of moisture content, barrel temperature, and screw speed on specific quality metrics. A composite quality index (CQI) was calculated as the equally weighted average of the standardized (z-score) values of WHC, integrity index, NSI, springiness, and cohesiveness, as shown in Equation (8). Chewiness and vertical cutting strength were not included in the CQI because they represent firmness-related texture attributes that may exhibit trade-offs with the hydration-related quality attributes included in the CQI. Therefore, they were analyzed separately using supplementary exploratory multiple regression analyses to provide complementary insights into texture-specific responses to extrusion processing variables.

(8) $$CQI=\frac{Z_{WHC}+Z_{II}+Z_{NSI}+Z_{Springness}+Z_{Cohesiveness}}{5}$$

*Z* represents the standardized (z-score) value of each quality parameter. Chewiness and vertical cutting strength were evaluated independently, as these textural attributes may reflect trade-offs in firmness rather than consistent improvements in overall quality. Standardized regression coefficients (β), coefficients of determination (R^2^), F-values, and statistical significance (*p*-values) were used to interpret the exploratory regression models.

## 3. Results

### 3.1. Appearance

**Figure 2:**
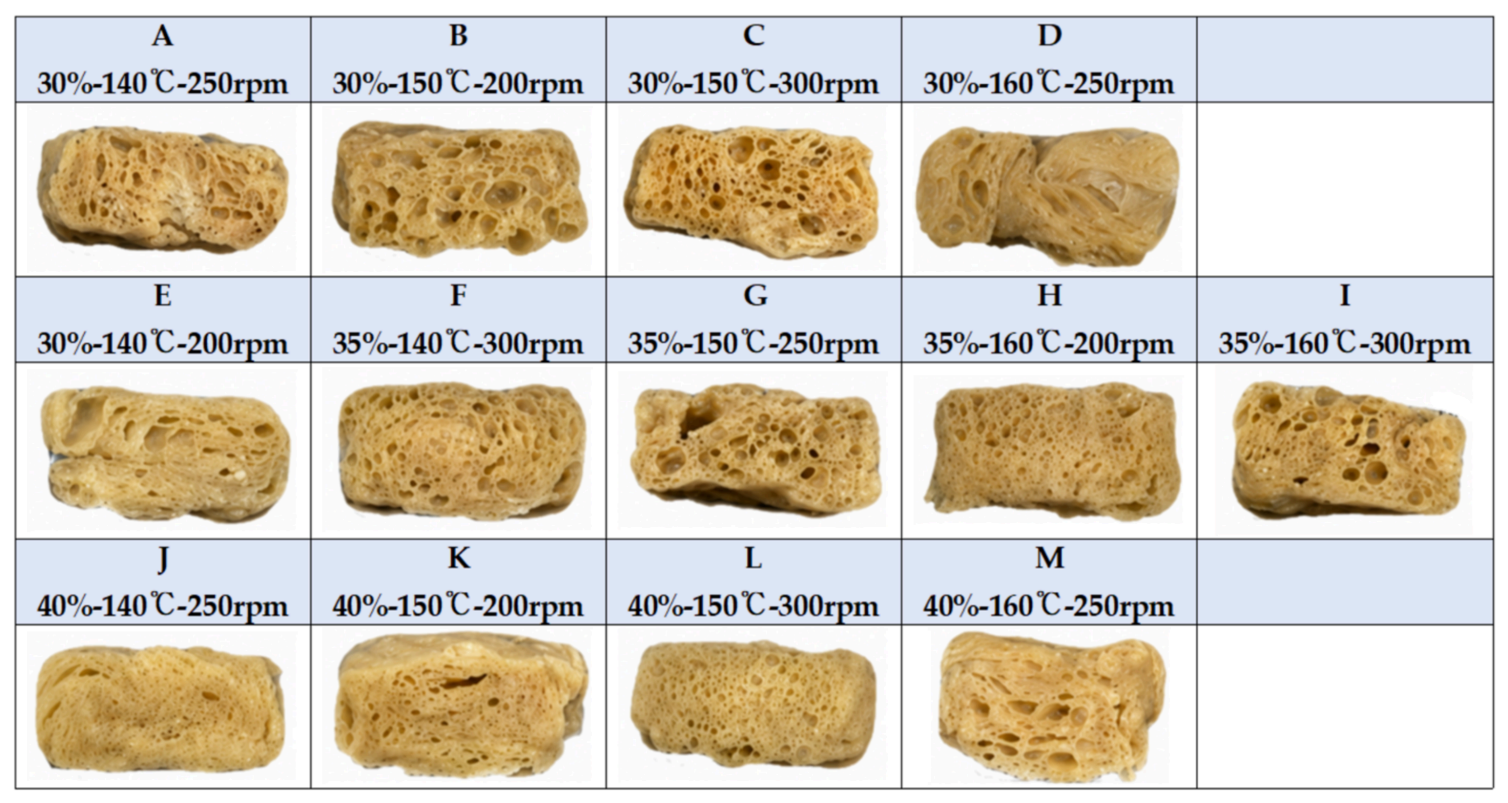
Cross-sections of low-moisture meat analogs made from isolated rice protein under different process parameters.

Figure 2 shows the cross-sectional images of isolated rice protein-enhanced low-moisture meat analogs (IRPE-LMMAs) produced under different extrusion conditions. The internal morphology of the samples differed depending on the processing variables, and the most apparent visual difference was observed with changes in feed moisture content. Samples produced at lower moisture levels showed a relatively open and porous structure, with larger and more clearly visible voids in the cross-section. In contrast, samples prepared at higher moisture levels appeared more compact, and the number of visible pores tended to decrease. These visual differences suggest that moisture content influenced the expansion behavior and structural development of IRPE-LMMAs during low-moisture extrusion.

The porous structure of extruded meat analogs is closely related to hydration behavior, rehydration rate, structural integrity, and textural quality [20]. In low-moisture extrusion, water acts not only as a plasticizer but also as a factor that modifies melt viscosity, protein mobility, and the degree of expansion at the die exit [21]. At higher feed moisture levels, this enhanced plasticization and lower melt viscosity lead to a significant reduction in elastic recovery as the melt exits the die, thereby suppressing overall radial expansion. Furthermore, higher moisture contents modify the thermodynamic conditions at the die exit by reducing the rate of flash evaporation of superheated water. This altered thermodynamic state restricts the rate of bubble nucleation and vapor cell growth, while simultaneously accelerating the collapse of unstable cell walls prior to matrix solidification. Consequently, these combined mechanisms suppress macro expansion and lead to a much denser extrudate matrix with fewer visible voids. In contrast, at lower moisture contents, the high viscosity and pronounced elastic recovery, coupled with rapid flash evaporation, promote bubble expansion and result in a highly porous internal structure with larger voids [22,23].

Changes in moisture content had a more pronounced effect on the visible pore structure than variations in barrel temperature and screw speed. Although barrel temperature and screw speed can affect protein disruption, melt flow, and shear history during extrusion, their visual effects on the cross-sectional structure were not as clear as those of moisture content in this study. Therefore, the appearance results indicate that feed moisture content was the primary processing factor affecting the visible internal morphology of IRPE-LMMAs. However, because the present observation was based on cross-sectional images, further quantitative image analysis would be required to confirm changes in pore number, pore size distribution, and cell-wall thickness.

### 3.2. Water Holding Capacity (WHC)

The water-holding capacity (WHC) of the IRPE-LMMA samples exhibited variations in accordance with the extrusion parameters, as presented in Table 2. WHC is an important quality attribute of low-moisture meat analogs because it reflects the ability of dried textured products to absorb and retain water during rehydration. In this study, samples produced at higher moisture content generally showed greater WHC than those prepared at lower moisture content. This result indicates that feed moisture played an important role in determining the hydration behavior of IRPE-LMMAs after extrusion and drying.

The observed increase in WHC at elevated moisture levels may be related to changes in protein hydration, matrix formation, and internal structure during extrusion. The plasticizing effect of water in the protein-rich melt promotes molecular movement, thereby assisting in the structural development of the hydrated protein network [21]. After drying and rehydration, this network may provide more sites for water absorption and retention. Beyond simple protein denaturation, the complete gelatinization of starch components during extrusion disrupts their crystalline structures, thereby exposing numerous hydrophilic hydroxyl groups that actively bind free water molecules during rehydration [24]. Concurrently, the intense thermal and mechanical stresses facilitate the formation of insoluble protein aggregates stabilized by covalent disulfide cross-linking and non-covalent hydrophobic interactions, which directly optimizes the microcapillary pore distribution within the texturized matrix to trap water more effectively [25]. In addition, the denaturation and aggregation of soy protein, rice protein, and wheat gluten during extrusion may contribute to the construction of a three-dimensional matrix capable of retaining water within the structure [26].

Barrel temperature also appeared to influence WHC, although the effect was dependent on the combination of moisture content and screw speed. Higher barrel temperature can promote protein unfolding and aggregation, which may strengthen the protein matrix and improve water retention after rehydration [27]. Conversely, substantial thermal or mechanical treatment may also modify the structure differently depending on the moisture level. Therefore, the effect of barrel temperature should be interpreted together with feed moisture rather than as an independent factor.

The effect of screw speed on WHC showed greater variability across the experimental conditions tested. Increased screw speed may enhance shear-induced structural modification and affect pore formation, but the resulting WHC can vary depending on the balance between shear intensity, melt viscosity, and protein network development. Similar investigations into extrusion processing have reported that variations in operational conditions are capable of altering the water-holding capacity of textured protein products [28]. Overall, the WHC results suggest that feed moisture content was the most apparent factor affecting the rehydration capacity of IRPE-LMMAs, while barrel temperature and screw speed contributed in a condition-dependent manner.

### 3.3. Integrity Index

The integrity index of IRPE-LMMAs is summarized in Table 2. This parameter characterizes the fraction of structured residue that persists following hydration, thermal processing, dispersion, rinsing, and subsequent dehydration. A higher integrity index indicates greater resistance to structural breakdown during rehydration and mechanical dispersion, and it is therefore associated with improved structural stability of textured protein products [18,20].

In this study, the integrity index values changed within a relatively narrow range compared with other physicochemical properties. This suggests that all extrusion conditions produced a certain level of structural retention in IRPE-LMMAs. However, the response of the integrity index to processing variables was not completely linear. Moisture content appeared to affect structural stability differently depending on the combination of barrel temperature and screw speed. At relatively mild processing conditions, changes in moisture content did not produce a clear reduction in the integrity index. In contrast, under higher thermal or mechanical input, increased moisture content tended to reduce the integrity index, suggesting that excessive water availability may weaken the compact protein network formed during low-moisture extrusion.

This tendency may be explained by the role of moisture in modifying protein interactions and melt behavior. Under low-moisture conditions, protein molecules are exposed to stronger mechanical shear and may form a more compact and resistant network after denaturation and aggregation [29]. In the present formulation, the structural network is likely governed by the complementary functionalities of ISP, IRP and WG during extrusion. Thermal and mechanical energy induces the unfolding of ISP, promoting intermolecular associations through hydrophobic interactions and disulfide bond formation, whereas WG develops a continuous viscoelastic network that serves as the primary structural framework of the extrudate [30,31,32]. Although IRP exhibits relatively limited network-forming ability when processed alone because of its low solubility and compact molecular structure, it is expected to function as a complementary protein component within the developing protein (ISP, WG) matrix during extrusion, thereby contributing to the stabilization of the overall protein network [33,34]. Such interactions are likely to contribute to the formation of a more stable three-dimensional protein network, thereby influencing the structural integrity of IRPE-LMMAs. Therefore, the structural integrity of IRPE-LMMAs is likely governed not only by the extent of protein denaturation and aggregation but also by the cooperative interactions among ISP, IRP, and WG that occur during extrusion processing. This interpretation is further supported by previous studies reporting that low-moisture meat analogs formulated with ISP, WG, and IRP, as well as high-moisture meat analogs prepared from ISP, IRP, and CS, successfully developed stable structures with desirable physicochemical and textural characteristics after extrusion [15,35].

Despite the contribution of these complementary protein functionalities to network formation, increasing moisture content can plasticize the material, reduce melt viscosity, and increase molecular mobility [21]. Although these effects may improve hydration-related properties, they can also reduce the compactness of the final structure and make the matrix more susceptible to breakdown during the integrity index procedure. Similar relationships between extrusion-induced protein structure and integrity have been reported in previous studies on textured protein and meat analog systems [18,20,28].

The impacts of barrel temperature and screw speed were observed to be more complex than the influence exerted by moisture content. Increased barrel temperature may promote protein denaturation and network formation, but its effect on integrity depends on whether the resulting structure is sufficiently stabilized during extrusion and drying. Similarly, screw speed can increase shear input, but excessive or insufficient shear may lead to different structural outcomes depending on moisture level. Therefore, the integrity index results indicate that the structural stability of the IRPE-LMMAs was governed by the combined effects of moisture content, thermal treatment, and mechanical shear rather than by a single extrusion variable.

### 3.4. Nitrogen Solubility Index (NSI)

Table 2 shows that the nitrogen solubility index (NSI) of the IRPE-LMMAs is influenced by varying extrusion parameters. NSI represents the proportion of soluble nitrogen remaining in the extruded sample and can serve as an indirect indicator of protein denaturation, aggregation, and solubility changes during extrusion. In general, extrusion processing reduces protein solubility because thermal and mechanical stresses promote protein unfolding and aggregation. These structural changes expose hydrophobic regions and free sulfhydryl groups, facilitating the formation of insoluble aggregates stabilized by disulfide bonds and hydrophobic interactions [36]. Therefore, changes in NSI reflect the balance between protein structural modification and the retention of soluble protein fractions.

In this study, NSI tended to increase with increasing moisture content, although the magnitude of this change was influenced by barrel temperature and screw speed. Specifically, as shown in Table 2, NSI values at 30% moisture content ranged from 34.36% to 45.37%, whereas samples produced at 40% moisture content generally exhibited higher values ranging from 47.60% to 63.49%. This tendency suggests that higher feed moisture may have partially reduced the extent of protein aggregation during low-moisture extrusion. Water can increase protein hydration and molecular mobility, while also reducing melt viscosity and moderating the intensity of thermal and mechanical stress applied to the protein matrix [21]. As a result, samples produced at higher moisture levels may retain a greater proportion of soluble nitrogen after extrusion.

The NSI results showed a tendency opposite to that of the integrity index. While higher moisture content may increase nitrogen solubility, it may also weaken the compactness of the structured protein network [37]. This suggests that increased protein solubility does not necessarily indicate improved structural stability in low-moisture meat analogs. Rather, NSI should be interpreted together with integrity index and textural properties to understand the overall quality of IRPE-LMMA.

The influence of barrel temperature and screw speed on NSI was less consistent than the effect of moisture content. Barrel temperature did not show a uniform influence across all conditions, indicating that thermal effects on protein solubility were dependent on the moisture level and shear environment. Similarly, screw speed showed a relatively limited influence under certain processing conditions, but its effect varied depending on moisture content and barrel temperature. This condition-dependent response is consistent with previous extrusion studies showing that protein solubility is modulated by the combined action of moisture, temperature, and mechanical shear rather than by a single processing variable [19]. Such interactive behavior may be associated with changes in melt viscosity and shear intensity under different combinations of moisture content, barrel temperature, and screw speed, which can influence the balance between protein aggregation and the retention of soluble protein fractions by modifying protein mobility, interactions, and network formation during extrusion [25]. Notably, considerable variability in NSI values was observed across several treatments, as indicated by the relatively large standard deviations. This variability may be associated with structural heterogeneity among the extrudate samples or transient variations in material flow during extrusion. However, because these factors were not directly monitored, the exact source of the variability could not be conclusively determined. Overall, feed moisture content showed the clearest tendency to influence NSI in IRPE-LMMA, while the effects of barrel temperature and screw speed varied according to the specific combinations of extrusion processing conditions.

### 3.5. Texture Profile Analysis (TPA) and Cutting Strength

The texture profile analysis and cutting strength values of IRPE-LMMAs produced under different extrusion conditions are presented in Table 3. The texture of rehydrated low-moisture meat analogs is a vital quality parameter, primarily because it defines the structural perception, elasticity, and resistance of the product during consumption. In this study, the textural responses were influenced mainly by moisture content, while the influence exerted by barrel temperature and screw speed appeared less predictable under the tested conditions.

Springiness and cohesiveness generally increased as moisture content increased. This result suggests that higher feed moisture promoted the formation of a more elastic and cohesive matrix during extrusion. Water can enhance protein mobility and facilitate molecular rearrangement, allowing the protein components to form a continuous structure after thermal and mechanical treatment [38]. The presence of soy protein, rice protein, and wheat gluten may also have contributed to the development of an elastic network, particularly under conditions where sufficient moisture was available for protein hydration and interaction.

In contrast, chewiness tended to decrease as moisture content increased. This indicates that improved elasticity and cohesion did not necessarily lead to greater resistance to mastication. Chewiness is affected not only by springiness and cohesiveness but also by the maximum stress generated during compression [39]. Therefore, samples with higher moisture content may have formed a softer and more flexible structure, resulting in lower chewiness despite their relatively high springiness and cohesiveness.

Cutting strength showed a similar tendency to chewiness. Both vertical and parallel cutting strengths were generally higher in samples produced at lower moisture content and lower in samples produced at higher moisture content. This result indicates that lower moisture conditions favored the formation of a firmer and more resistant structure, whereas higher moisture conditions reduced structural firmness. Higher moisture content likely reduced melt viscosity and weakened protein–protein interactions during extrusion, resulting in a less dense protein network and consequently lower cutting strength after drying [25].

The effects of barrel temperature and screw speed on textural properties were found to be inconsistent. Although these variables can influence protein denaturation, shear history, and structural development, their effects appeared to depend on the moisture level and the specific texture parameter considered [40]. Therefore, the texture results suggest that moisture content was the primary factor controlling the balance between elasticity and firmness in IRPE-LMMAs. Higher moisture content improved springiness and cohesiveness, whereas lower moisture content contributed to greater chewiness and cutting resistance. These findings indicate a clear texture-related trade-off that should be considered when selecting extrusion conditions for IRPE-LMMA production.

### 3.6. Optimization of Process Variables

Response surface methodology was employed to investigate the effects of moisture content, barrel temperature, and screw speed on the chewiness of IRPE-LMMAs. Chewiness was selected as the key response variable because it is one of the most representative textural attributes reflecting the eating quality of low-moisture meat analogs. Detailed analysis of variance for the established quadratic model is summarized in Table 4.

The resulting regression model for chewiness demonstrated statistical significance, confirming that the chosen extrusion parameters effectively accounted for the variations observed within the experimental domain. Furthermore, the lack-of-fit was found to be non-significant, validating the suitability of the fitted model for modeling the response surface. Chewiness was substantially influenced by various model terms, including the linear effects of moisture content, barrel temperature, and screw speed; interaction effects (moisture content/barrel temperature and barrel temperature/screw speed); and the quadratic contributions of moisture content and screw speed. These findings demonstrate that chewiness is governed not only by independent processing factors but also by their synergistic and nonlinear interactions.

**Figure 3:**
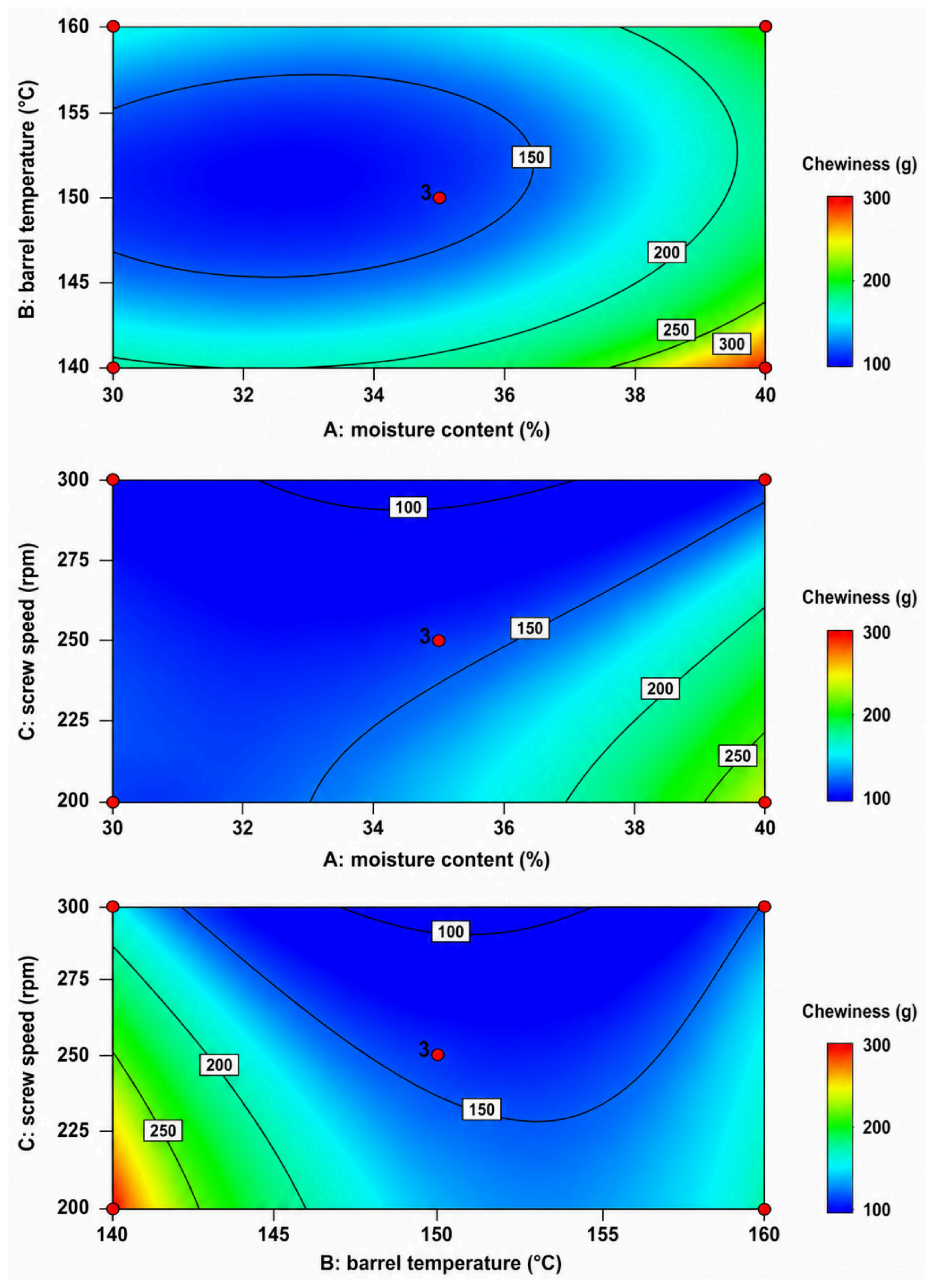
Graphical representation of response surface contours depicting the impact of barrel temperature, moisture content, and screw speed on the chewiness of IRPE-LMMAs.

The contour plots in Figure 3 show the response surface patterns for chewiness as affected by the extrusion variables. In general, chewiness decreased as moisture content increased, indicating that higher feed moisture produced a softer and less resistant structure [41]. This result is consistent with the texture profile analysis, where higher moisture content was associated with lower chewiness and cutting strength. Chewiness was also affected by barrel temperature and screw speed, though these impacts fluctuated based on moisture content levels and the interactions among processing parameters.

According to the response surface analysis, the optimized processing window for achieving desirable chewiness and structural quality was established within the design space of 39% to 40% moisture content, 141 °C to 144 °C barrel temperature, and 205 rpm to 215 rpm screw speed. Mechanistically, this specific combination achieves an ideal balance among key physical and chemical phenomena within the extruder barrel. The higher moisture range (39% to 40%) lowers melt viscosity to ensure proper flowability while preventing excessive shear degradation of the molecular chains. Concurrently, the moderate barrel temperature range (141 °C to 144 °C) may promote protein unfolding and further facilitate intermolecular interactions without causing excessive thermal degradation. Furthermore, the moderate screw speed (205 rpm to 215 rpm) delivers controlled mechanical shear, facilitating effective molecular alignment and intermolecular cross-linking. Consequently, these coordinated thermodynamic and mechanical conditions foster the formation of a continuous, highly elastic protein network, yielding the optimum chewiness required for low-moisture meat analogs. Therefore, these conditions should be interpreted as practical processing conditions for chewiness optimization, rather than as universal optimum conditions for all quality attributes of IRPE-LMMAs. Because this study focused on chewiness as the representative response variable, the optimization was not intended to simultaneously optimize all physicochemical and textural properties. Future studies employing multi-objective optimization approaches could provide a more comprehensive framework for simultaneously optimizing multiple quality attributes.

### 3.7. Exploratory Multiple Regression Analysis of Integrated Quality and Texture-Specific Properties

In addition to response surface optimization focused on chewiness, exploratory multiple regression analyses were conducted using Composite Quality Index (CQI), chewiness, and vertical cutting strength as dependent variables (Table 5). The regression model for CQI was statistically significant (F = 5.491, R^2^ = 0.600), indicating that extrusion variables explained 60.0% of the variation in integrated physicochemical quality. Among the tested variables, moisture content showed the strongest positive effect on CQI (β = 0.728, *p* < 0.01), confirming that moisture content was the dominant factor improving integrated physicochemical quality. However, barrel temperature and screw speed exhibited positive but non-significant effects.

This finding suggests that hydration-related quality attributes were predominantly governed by feed moisture under the tested extrusion conditions, whereas barrel temperature and screw speed made comparatively limited contributions to the integrated physicochemical quality represented by the CQI.

For chewiness, the regression model was also significant (F = 3.796, R^2^ = 0.509). Moisture content showed a negative association with chewiness (β = −0.377), while barrel temperature (β = 0.398, *p* < 0.1) and screw speed (β = −0.456, *p* < 0.1) demonstrated marginal effects, suggesting that textural firmness may be influenced by multiple process variables rather than moisture content alone. Unlike the CQI, chewiness appeared to be influenced by a combination of extrusion variables, indicating that firmness-related texture is governed by more complex processing conditions.

Vertical cutting strength analysis similarly showed a significant model fit (F = 3.977, R^2^ = 0.520), with moisture content exerting a significant negative effect (β = −0.612, *p* < 0.05), suggesting that increasing moisture content may weaken structural firmness. Barrel temperature and screw speed showed comparatively small and non-significant effects. This contrasting response further highlights the trade-off between hydration-related functionality and structural firmness during extrusion processing.

These findings collectively demonstrate that moisture content is the dominant driver of integrated physicochemical quality enhancement, whereas firmness-related properties responded differently to the extrusion variables. Therefore, process optimization for IRPE-LMMAs should not be interpreted solely as maximizing overall quality, but rather as balancing hydration-related functionality with meat-like textural properties.

## 4. Conclusions

The impact of moisture content, barrel temperature, and screw speed on the physicochemical and textural attributes of isolated rice protein-enhanced low-moisture meat analogs, fabricated via low-moisture extrusion, was comprehensively evaluated in this study. Among the tested processing variables, moisture content showed the most apparent influence on product quality. Higher moisture content tended to improve hydration-related and elastic properties, including water-holding capacity, nitrogen solubility index, springiness, and cohesiveness. However, increased moisture content also reduced chewiness and cutting strength, indicating a trade-off between hydration functionality and structural firmness.

Response surface methodology based on chewiness identified a practical processing window within the experimental range of 39–40% moisture content, 141–144 °C barrel temperature, and 205–215 rpm screw speed. Therefore, these conditions should be interpreted as chewiness-oriented processing conditions rather than universal optimum conditions for all quality attributes. Supplementary exploratory regression analysis further suggested that moisture content was the primary determinant of the composite quality index, indicating that hydration-related quality attributes were predominantly governed by feed moisture under the tested extrusion conditions. However, firmness-related responses should be considered separately because they followed a different response pattern. Overall, these findings suggest that optimization of IRPE-LMMAs should balance hydration-related functionality, elasticity, and structural firmness according to the intended product application. Because motor load, die pressure, and specific mechanical energy were not recorded during extrusion in the present study, future studies should include these process parameters to further clarify the relationships between operating conditions, mechanical energy input, pressure development, and the resulting quality characteristics of IRPE-LMMAs.

## Figures and Tables

**Table 1:** Experimental runs and processing conditions based on the Box–Behnken design.

Run Order	X_1_	X_2_	X_3_
	Moisture Content (%)	Barrel Temperature (°C)	Screw Speed (rpm)
1	30	140	250
2	30	150	200
3	30	150	300
4	30	160	250
5	35	140	200
6	35	140	300
7	35	150	250
8	35	150	250
9	35	150	250
10	35	160	200
11	35	160	300
12	40	140	250
13	40	150	200
14	40	150	300
15	40	160	250

Runs 7, 8, and 9 represent randomized center-point replicates.

**Table 2:** Physicochemical properties of IRPE-LMMAs at various extrusion conditions.

Run Order	Water Holding Capacity (%)	Integrity Index (%)	Nitrogen Solubility Index (%)
1	165.79 ± 10.91 f	64.73 ± 8.40 cde	34.91 ± 4.07 b
2	162.50 ± 11.94 f	68.95 ± 5.33 e	34.36 ± 5.76 b
3	222.50 ± 4.93 def	73.70 ± 3.03 a	41.41 ± 8.78 ab
4	299.02 ± 10.17 f	68.35 ± 6.20 a	45.37 ± 3.66 ab
5	298.26 ± 11.95 f	73.54 ± 4.17 cde	31.42 ± 2.91 b
6	274.61 ± 15.31 bc	73.55 ± 2.87 bc	48.77 ± 16.12 ab
7	330.04 ± 17.19 bc	69.99 ± 2.31 cde	41.50 ± 2.54 ab
8	330.81 ± 18.62 bc	73.82 ± 1.00 bcd	52.28 ± 19.42 ab
9	327.88 ± 5.18 c	73.05 ± 2.91 cde	39.31 ± 4.07 ab
10	243.98 ± 10.12 de	68.10 ± 2.12 de	45.16 ± 18.65 ab
11	364.07 ± 4.29 a	68.41 ± 4.14 cde	59.74 ± 26.41 ab
12	299.60 ± 16.58 d	68.55 ± 3.99 cde	47.60 ± 16.49 ab
13	327.04 ± 15.18 ef	71.50 ± 3.07 e	63.49 ± 24.67 a
14	334.51 ± 22.89 a	70.58 ± 2.89 cde	50.10 ± 14.18 ab
15	398.71 ± 21.44 b	69.23 ± 3.50 ab	51.09 ± 13.42 ab

Values with different letters in the same column indicate significant differences (*p* < 0.05) by Duncan’s multiple range test.

**Table 3:** Texture profile analysis and cutting strength of isolated rice protein-enhanced low-moisture meat analog according to process variables.

Run Order	Texture Profile Analysis			Cutting Strength (g cm^−2^)	
	Springiness (%)	Cohesiveness (%)	Chewiness (g)	Vertical Direction	Parallel Direction
1	80.75 ± 10.44 ^e^	77.97 ± 6.75 ^f^	252.66 ± 59.44 ^abcd^	643.88 ± 135.20 ^b^	445.54 ± 64.42 ^a^
2	88.60 ± 4.39 ^abcd^	84.09 ± 4.34 ^de^	304.46 ± 146.54 ^ab^	779.02 ± 166.00 ^a^	444.13 ± 156.28 ^a^
3	85.81 ± 3.98 ^cde^	85.59 ± 1.33 ^bcde^	163.37 ± 113.08 ^cd^	400.43 ± 42.80 ^defgh^	252.09 ± 31.67 ^cde^
4	86.60 ± 3.96 ^bcde^	82.68 ± 4.88 ^ef^	336.16 ± 226.18 ^a^	653.90 ± 168.05 ^b^	337.81 ± 81.88 ^b^
5	91.56 ± 9.94 ^abc^	87.07 ± 6.85 ^abcde^	134.54 ± 73.96 ^d^	485.72 ± 101.61 ^cde^	291.42 ± 41.33 ^bcd^
6	93.08 ± 3.35 ^ab^	90.90 ± 4.59 ^ab^	126.98 ± 28.87 ^d^	475.37 ± 78.91 ^cdef^	306.17 ± 64.91 ^bc^
7	90.83 ± 3.01 ^abc^	87.41 ± 1.77 ^abcde^	130.24 ± 81.63 ^d^	386.69 ± 55.71 ^efgh^	252.35 ± 40.69 ^cde^
8	83.38 ± 9.78 ^de^	81.96 ± 12.70 ^ef^	154.47 ± 87.04 ^cd^	377.05 ± 34.15 ^fgh^	274.10 ± 46.04 ^bcde^
9	88.17 ± 6.49 ^abcd^	86.98 ± 3.69 ^abcde^	141.80 ± 62.73 ^d^	373.90 ± 10.76 ^fgh^	257.31 ± 29.92 ^cde^
10	90.81 ± 3.47 ^abc^	89.96 ± 2.21 ^abc^	270.07 ± 155.20 ^abc^	489.47 ± 77.16 ^cd^	254.61 ± 38.86 ^cde^
11	90.42 ± 3.85 ^abc^	89.24 ± 1.50 ^abcd^	140.41 ± 61.41 ^d^	424.38 ± 89.51 ^cdefg^	244.56 ± 44.86 ^cde^
12	91.83 ± 2.40 ^abc^	86.95 ± 2.36 ^abcde^	183.66 ± 87.99 ^cd^	525.17 ± 61.10 ^c^	314.67 ± 51.55 ^bc^
13	92.39 ± 3.35 ^abc^	91.31 ± 1.67 ^a^	205.67 ± 96.55 ^bcd^	360.32 ± 50.20 ^gh^	204.93 ± 27.60 ^e^
14	93.85 ± 1.51 ^a^	91.57 ± 0.96 ^a^	153.39 ± 56.23 ^cd^	303.19 ± 33.38 ^h^	223.02 ± 28.58 ^de^
15	86.60 ± 10.44 ^bcde^	84.78 ± 2.76 ^cde^	240.15 ± 94.21 ^abcd^	449.77 ± 60.50 ^cdefg^	225.05 ± 34.40 ^de^

Values with different letters in the same column indicate significant differences (*p* < 0.05) by Duncan’s multiple range test.

**Table 4:** Analysis of variance of the regression model for chewiness optimization.

Source	*p*-Value
Model	0.0006
X_1_— moisture content	0.0010
X_2_—barrel temperature	0.0005
X_3_—screw speed	0.0012
X_1_X_2_	0.0091
X_1_X_3_	0.4031
X_2_X_3_	0.0300
X_1_^2^	0.0054
X_2_^2^	0.2383
X_3_^2^	0.0002
Lack of Fit	0.3746
R^2^	0.9834
Adj R^2^	0.9535
C.V. %	7.55

**Table 5:** Exploratory multiple regression analysis of integrated quality and texture-specific properties.

Dependent Variable	M.C (%)	B.T (°C)	S.S (rpm)	F-Value	R^2^
Composite Quality Index (CQI)	0.728 ***	0.161	0.210	5.491	0.600
Chewiness	−0.377	0.398 *	−0.456 *	3.796	0.509
Vertical Cutting Strength	−0.612 **	−0.082	−0.373	3.977	0.520

Values represent standardized regression coefficients (β) from exploratory multiple regression models. Composite Quality Index (CQI) was calculated as the mean of the standardized values of WHC, integrity index, NSI, springiness, and cohesiveness. Chewiness and vertical cutting strength were analyzed separately to evaluate texture-specific trade-offs. * *p* < 0.1, ** *p* < 0.05, *** *p* < 0.01.

## Data Availability

The original contributions presented in this study are included in the article. Further inquiries can be directed to the corresponding author.
